# Adolescent's Educational Status and Reasons for Dropout from the School

**DOI:** 10.4103/0970-0218.40885

**Published:** 2008-04

**Authors:** B Maithly, Vartika Saxena

**Affiliations:** Rural Development Institute, Himalayan Institute of Medical Sciences, Doiwala, Dehradun

## Introduction

Adolescence is a period of acquiring new capacities. It is not only a time of opportunity but also of vulnerability to risky behavior, which can have life-long consequences, especially on education, career, and health.

Teachers play an important role in providing information and advice to the adolescents. Spectrum of information can range from routine education to career guidance and health advice. Often teachers play a role of counselor also for the students. Currently, proper system of professional counseling is lacking in our society and wherever they do exist, they are few and beyond the approach of the common person. School is the place where adolescents get opportunity to share many personal issues with their peers. Many queries get easily resolved, merely by having discussion with school friend or teachers, which otherwise may be difficult to get resolved at home. Those adolescents who unfortunately do not get chance to attend the schools during these formative years, may fail to spot such golden opportunities and lag behind in the support which they would have easily attained by just attending the school. This research paper attempts to assess the educational status of adolescent and to analyze the reasons for dropout from the school so that effective strategies can be developed for improving the situation.

## Materials and Methods

This study was carried out in the three districts of the Uttaranchal Viz. Dehradun, Nainital, and Udhamsingh Nagar. Two blocks in each district were selected for the study. Adolescents of age group of 13 to 19 were included for the study. A two-stage stratified sampling was used for arriving on the sampling unit. On an average, 20 households were selected for interviewing in each selected primary sampling unit. A total of 3,980 adolescents were covered in all the six blocks. Data were collected by interview method on pre-tested questionnaire. Informed consent was taken from the entire adolescent before interviewing them.

## Results

Total 3,980 adolescents were covered in six blocks of three districts of Uttaranchal. Overall literacy status was found very high (90%). Although, more than twice adolescent girls (14%) were found to be illiterate than boys (6%). Total 34% adolescents were found to have dropped from school, amongst them 41% were girls and 27% were boys.

The main reason for dropping out was financial difficulties for both girls and boys. Besides the financial reasons 31% boys and 13% girls reported that they are just not interested in further studies. A total of 28% girls said that their family and relatives did not approve their further continuation of the studies. A total of 9% girls and 1% boys reported lack of education facility in the nearby village as the reason for dropping out. Lack of quality education, imposition of parents choices upon adolescents, lack of privacy, and toilet facilities for girls in school and security reasons were few other reasons cited by adolescent for dropping out.

Out of 1,372 adolescents who dropped their studies, 73% adolescents expressed their wish for continuation of studies. Out of this, 46.2% were the girls and 26.4% were the boys.

## Discussion

Adolescence is the phase of turbulence where a child goes through many physical, psychological, and emotional changes. If the adolescent is attending the school during this phase, it is not only good for educational attainments but also for getting opportunity for sharing many turbulent thoughts with their peers and sometime with teachers also.

In this study, 6% boys and 14% girls were found to be illiterate, which is far less than the national figure of 20% and 44% and a positive indication for the state. Study indicated 73% male and 59% of female attending the school in the age group of 13 to 19. Proportionately lower figures were reported by Central Statistical Organization, youth India (1998-99) as 65.2% for male and 49.08% for female for the age group of 11 to 14 years. Apparently, the percentage of females attending the school has increased more than males in recent years but still they lag behind that had to be seen as matter of concern. The drop out rates even at primary level is more for the girls than the boys. The main reason for girls remaining behind is the attitude of the parents. Other reasons are the burden of sibling care, domestic work, physical and sexual insecurity, parental education level etc., indicating the need of interventions in the family and social domains. Of course, the school conditions need to be made more girl-friendly, like appointment of more female teachers, provision of separate toilet for them, and doing away the physical punishment. A further reduction in gaps between males and females in enrolment and retention can be achieved by understanding dynamic of interactions among household work, paid labor, and female education. These factors need to be properly addressed for not only improving the female literacy rate but also reduction in the overall dropout rates.

**Table 1:** Reasons for dropping out from the school

Reasons for dropping out	Male%	Female%	Total%
Not interested in studying	197 (31)	127 (13)	324 (20)
Family/relatives did not approve	37 (6)	274 (28)	311 (19)
Financial reasons	253 (40)	290 (30)	543 (34)
Required for household work	16 (3)	71 (7)	87 (5)
Facility for further study does not exist nearby village	7 (1)	88 (9)	95 (6)
Others	128 (20)	114 (12)	242 (15)
Total	638	964	1,602
